# Magnetic Resonance Imaging after Completion of Neoadjuvant Chemotherapy Can Accurately Discriminate between No Residual Carcinoma and Residual Ductal Carcinoma *In Situ* in Patients with Triple-Negative Breast Cancer

**DOI:** 10.1371/journal.pone.0149347

**Published:** 2016-02-11

**Authors:** Seho Park, Jung Hyun Yoon, Joohyuk Sohn, Hyung Seok Park, Hee Jung Moon, Min Jung Kim, Eun-Kyung Kim, Seung Il Kim, Byeong-Woo Park

**Affiliations:** 1 Department of Surgery, Yonsei University College of Medicine, Seoul, Republic of Korea; 2 Department of Radiology, Research Institute of Radiological Science, Severance Hospital, Yonsei University College of Medicine, Seoul, Republic of Korea; 3 Division of Medical Oncology, Department of Internal Medicine, Yonsei University College of Medicine, Seoul, Republic of Korea; University of Chicago, UNITED STATES

## Abstract

**Background:**

The accurate evaluation of favorable response to neoadjuvant chemotherapy (NCT) is critical to determine the extent of surgery. We investigated independent clinicopathological and radiological predictors to discriminate no residual carcinoma (ypT0) from residual ductal carcinoma *in situ* (ypTis) in breast cancer patients who received NCT.

**Patients and Methods:**

Parameters of 117 patients attaining pathological complete response (CR) in the breast after NCT between January 2010 and December 2013 were retrospectively evaluated by univariate and multivariate analyses. All patients underwent mammography, ultrasound, and magnetic resonance imaging (MRI) before and after NCT.

**Results:**

There were 67 (57.3%) patients with ypT0. These patients were associated with hormone receptor-negative status, human epidermal growth factor receptor-2 (HER2)-negative tumors, and a higher likelihood of breast-conservation surgery. Baseline mammographic and MRI presentation of the main lesion, absence of associated microcalcifications, shape, posterior features, and absence of calcifications on ultrasound were significantly associated with ypT0. CR in mammography, ultrasound, or MRI after NCT was also related to ypT0. By multivariate analysis, independent predictors of ypT0 were the triple-negative subtype [Odds ratio (OR), 4.23; 95% confidence interval (CI), 1.11–16.09] and CR in MRI after NCT (OR, 5.23; 95% CI, 1.53–17.85). Stratified analysis by breast cancer subtype demonstrated that MRI well predicted ypT0 in all subtypes except the HER2-positive subtype. In particular, of 40 triple-negative subtypes, 22 showed CR in MRI and 21 (95.5%) were ypT0 after NCT.

**Conclusion:**

Among imaging modalities, breast MRI can potentially distinguish between ypT0 and ypTis after NCT, especially in patients with triple-negative breast cancer. This information can help clinicians evaluate tumor response to NCT and plan surgery for breast cancer patients of all subtypes except for those with HER2-enriched tumors after NCT.

## Introduction

Neoadjuvant chemotherapy (NCT) is now commonly considered for breast cancer patients who are potential candidates for adjuvant chemotherapy and it has been reported to have similar oncologic outcomes to adjuvant chemotherapy [1]. In addition, NCT increases the chances of successful breast-conservation surgery, facilitates tumor biology research, and most importantly, provides information about prognosis [1–3]. For these advantages to be of use in real clinical practice, accurate evaluation of response during NCT and preoperative assessment of residual tumor burden through imaging modalities are critical for planning the extent of surgery and for predicting prognosis. Recently, a meta-analysis suggested that breast magnetic resonance imaging (MRI) showed good performance in predicting pathologic complete response (pCR) after NCT [4].

Residual ductal carcinoma *in situ* (DCIS) components of breast cancer after NCT are considered as pCR; however, surgery is differently planned if these components are of no residual invasive and *in situ* carcinoma (ypT0). Obtaining clear resection margins with accurate preoperative evaluation helps decrease operation time and reduces the chances of repeating surgery or early local recurrence. Chen et al. [5] demonstrated that positive cavity margin was the only independent predictor for local-regional failure in patients treated with NCT before breast-conservation surgery according to univariate and multivariate analysis. Most clinicians usually plan the extent of surgery to achieve negative resections based on radiological examinations and clinicopathological parameters. However, it has not been established which parameters should have higher priority in daily practice.

In our review of previous literatures, there was only one article that dealt with discriminating ypT0 from residual DCIS in the breast after NCT [6]. In that study, the dynamic contrast-enhanced MRI was reported to show good performance for distinguishing between lesions with or without residual DCIS in breast cancer patients who demonstrated no residual invasive cancer after NCT [6]. However, the study sample was limited, including only 15 cases of residual *in situ* carcinoma. It is therefore difficult to generalize their results to other samples, or to analyze clinicopathological factors such as breast cancer phenotype, Ki-67 levels, or the use of human epidermal growth factor receptor-2 (HER2) targeted therapy [7,8]. Thus, more comprehensive studies are necessary to determine the potential of MRI alongside future analyses of clinicopathological findings of breast cancer patients who receive NCT.

The aim of this study was to investigate independent clinicopathological and radiological characteristics, including breast cancer subtypes, in order to discriminate between ypT0 and residual DCIS alone (ypTis) on final pathology in breast cancer patients who responded well to NCT.

## Patients and Methods

### Patient selection

A total of 163 patients who achieved pCR in the breast after receiving NCT and who subsequently underwent definitive surgery of the breast and axilla from January 2010 to December 2013 at the Severance Hospital of Yonsei University College of Medicine, Seoul, Republic of Korea were retrospectively selected. All patients in the study cohort were histologically confirmed to have primary invasive breast carcinoma at initial presentation. After therapeutic surgery, permanent pathologic findings of the breast for all patients were reported as no residual invasive and *in situ* carcinoma (ypT0) or residual *in situ* carcinoma alone (ypTis), irrespective of pathologic nodal stage (ypNany). Forty-six (28.2%) patients who did not undergo mammography, ultrasound, and breast MRI both prior to and after NCT were excluded from analysis. Therefore, 117 patients were finally included in our study.

NCT regimens were mainly composed of 4 cycles of anthracycline plus cyclophosphamide (AC) followed by 4 cycles of docetaxel (T) every 3 weeks in 91 (77.8%) patients. Twelve (10.3%) patients received AC followed by T plus TS-1. Of the remaining 14 (12.0%) patients, 8 were treated with 6 cycles of T plus carboplatinum with bevacizumab and 2 received paclitaxel plus carboplatinum. Each patient went through one of four regimens: four cycles of AC, 6 cycles of TAC, T plus carboplatinum with trastzumab, or paclitaxel plus trastzumab. This study was approved by the Institutional Review Board of Severance Hospital, Yonsei University Health System, Seoul, Republic of Korea (IRB No. 4-2015-0247). The requirement for written informed consent was waived and patient information was anonymized and de-identified prior to analysis.

### Clinicopathological characteristics

Clinicopathological information, including expression of the estrogen receptor (ER), progesterone receptor (PR), HER2, and Ki-67, was obtained through reviews of medical records and pathology reports. Tumors with ≥1% nuclear-stained cells by immunohistochemistry of core needle biopsy specimens prior to NCT were considered positive for hormone receptors (HRs) according to the American Society of Clinical Oncology/College of American Pathologists (ASCO/CAP) guidelines [9]. HER2 staining was scored as 0, 1+, 2+, or 3+ according to ASCO/CAP guidelines [10]. In cases with HER2 2+ results, fluorescence *in situ* hybridization (FISH) was performed using a PathVysion *HER2* DNA Probe Kit (Vysis, Downers Grove, IL, USA) and HER2 gene amplification was defined with a HER2 gene/chromosome 17 copy number ratio ≥2.0 according to ASCO/CAP guidelines [10]. HER2 was considered positive with immunohistochemistry scores of 3+ or gene amplification by FISH. Ki-67 levels were scored by counting the number of positively stained nuclei and were expressed as a percentage of total tumor cells.

Breast cancer subtypes were categorized by HRs and HER2 expression as follows: HRs+/HER2-, ER-positive or PR-positive, and HER2-negative; HRs+/HER2+, ER-positive or PR-positive, and HER2-positive; HRs-/HER2+, ER-negative, PR-negative, and HER2-positive; HRs-/HER2-, ER-negative, PR-negative, and HER2-negative, a subtype also known as triple-negative breast cancer.

### Interpretation and analysis of imaging study

A radiologist (MJK) with more than 10 years of experience specializing in breast imaging interpreted mammography, ultrasound, and MRI images before and after NCT while blinded to clinicopathological information. Data on mammographic factors were collected by reviewing mammography before and after NCT and the mammographic factors reviewed were as follows. For mammography before NCT, tumor size (the largest diameter on mammography), the extent of the tumor [single and multiple: the presence of two of more tumor foci within a single quadrant of the breast (multifocal) or within different quadrants of the same breast (multicentric)], the presentation pattern of the main lesion (mass alone, the presence of microcalcifications regardless of mass, and non-visualization on mammography), the presence of associated microcalcifications for the main lesion, and other imaging characteristics of the main lesion (shape, margin, and density) and breast parenchymal patterns classified with the Breast Imaging Reporting and Data System (BI-RADS) by the American College of Radiology were studied [11]. For mammography after NCT, the mammographic factors studied for the presentation of the main lesion were as follows; complete response including cases undetected on mammography before NCT and residual mass or microcalcifications.

Ultrasonographic factors were reviewed and categorized as follows: For ultrasound before NCT, the tumor size was defined as the largest diameter on ultrasound and imaging findings of the main lesion were classified with BI-RADS [11]. Complete response or residual disease after NCT was also determined for ultrasound.

MRIs were reviewed before and after NCT and tumor size was defined as the largest diameter on the second post-contrast subtracted image. Background parenchymal enhancement was categorized into one of four levels (1. minimal, 2. mild, 3. moderate, and 4. marked). The type of lesion presented, the shape of the main lesion, the margin, the internal enhancement pattern, and the time-intensity curve (washout, plateau, and persistent) were assessed [11]. The time-intensity curve was evaluated using an automated software program (CADstream, Merge Healthcare, Chicago, IL, USA). The presence of intratumoral necrosis, fibrosis, perilesional edema, and the signal intensity of the lesion were evaluated with T2-weighted images (T2WI). Residual tumors were assessed on MRI after NCT. An enhancing area distinct from the background parenchymal enhancement was considered to indicate the presence of residual tumors. The absence of a distinct enhancing area was considered to indicate complete response to chemotherapy.

### Statistical analyses

Differences between the groups according to clinicopathological parameters were evaluated using the chi-square test. Fisher’s exact test was used when appropriate. The independent two sample *t*-test was used to compare the means of continuous numerical data. The predictive value of imaging modality for the detection of residual DCIS at the time before surgery was analyzed by receiver operating characteristic (ROC) curve analysis with calculated area under the ROC curve (AUC). A logistic regression analysis was used to investigate independent parameters including breast cancer subtype associated with ypT0 after completion of NCT. The Cochran-Mantel-Haenszel test was used in stratified analyses according to breast cancer subtype to explore the relationships between MRI findings after NCT and ypT0. All statistical tests were two-sided, and *p*-values <0.05 were considered statistically significant. The SPSS software version 20.0 (IBM Inc., Armonk, NY, USA) was used for all analyses.

## Results

Of 117 patients, 67 (57.3%) were ypT0 and 50 (42.7%) were ypTis after breast surgery. Mean age at diagnosis was 49.4 ± 9.7 years for the entire study sample. Table 1 presents clinicopathological characteristics according to presence of residual disease. There were no differences in clinical features, tumor burden at presentation, pathologic nodal status after NCT, histologic grade, Ki-67 proliferative index at diagnosis, or regimens of NCT between the two groups. Patients with ypT0 were more likely to have ER-negative, PR-negative, and HER2-negative tumors. Therefore, triple-negative breast cancer was significantly more common in the ypT0 group. Breast-conservation surgeries were more frequently performed in patients with ypT0.

**Table 1:** Clinicopathological findings of patients with ypT0 and ypTis in the breast after neoadjuvant chemotherapy.

Parameters	ypT0 (n = 67, %)	ypTis (n = 50, %)	*P*-value
Age (year)			
Mean ± SD	49.6 ± 10.4	49.1 ± 8.7	0.772^a^
≤40	13 (68.4)	6 (31.6)	0.283
>40	54 (55.1)	44 (44.9)	
Menopause			
Premenopause	35 (56.5)	27 (43.5)	0.850
Postmenopause	32 (58.2)	23 (41.8)	
BMI (kg/m^2^)			
<25	46 (57.5)	34 (42.5)	0.940
≥25	21 (56.8)	16 (43.2)	
Clinical tumor stage at presentation			
T1	26 (66.7)	13 (33.3)	0.340
T2	34 (53.1)	30 (46.9)	
T3-4	7 (50.0)	7 (50.0)	
Node status at presentation			
Negative	9 (64.3)	5 (35.7)	0.571
Positive	58 (56.3)	45 (43.7)	
Regimens of NCT			
AC followed by T	49 (73.1)	42 (84.0)	0.070
AC followed by T+TS1	6 (9.0)	6 (12.0)	
Others	12 (17.9)	2 (4.0)	
Pathologic node status after NCT			
ypN0	58 (60.4)	38 (39.6)	0.141
ypN1-3	9 (42.9)	12 (57.1)	
Histologic grade			
I/II	36 (56.2)	28 (43.8)	0.807
III	31 (58.5)	22 (41.5)	
ER			
Negative	43 (65.2)	23 (34.8)	0.050
Positive	24 (47.1)	27 (52.9)	
PR			
Negative	56 (62.2)	34 (37.8)	0.048
Positive	11 (40.7)	16 (59.3)	
HER2			
Negative	50 (68.5)	23 (31.5)	0.002
Positive	17 (38.6)	27 (61.4)	
Breast cancer subtype			
HRs+/HER2-	17 (51.5)	16 (48.5)	0.001
HRs+/HER2+	7 (36.8)	12 (63.2)	
HRs-/HER2+	10 (40.0)	15 (60.0)	
HRs-/HER2-	33 (82.5)	7 (17.5)	
Ki-67 before NCT (n = 105)			
≤15%	17 (45.9)	20 (54.1)	0.119
>15%	42 (61.8)	26 (38.2)	
Surgery			
Breast-conservation	46 (68.7)	21 (31.3)	0.004
Total mastectomy	21 (42.0)	29 (58.0)	

SD, standard deviation; BMI, body mass index; NCT, neoadjuvant chemotherapy; AC, anthracycline plus cyclophosphamide; T, docetaxel; ER, estrogen receptor; PR, progesterone receptor; HER2, human epidermal growth factor receptor 2; HRs, hormone receptors.

^a^Independent samples *t*-test.

Mammographic findings for patients with ypT0 and ypTis are compared in Table 2 and S1 Appendix. At initial presentation, the size, extent, shape, and margin of the main lesion, mammographic parenchymal pattern, and density did not differ between patients with ypT0 and ypTis. The baseline main tumor frequently presented as microcalcifications with or without mass in the ypTis group. Associated microcalcifications were more frequent in patients with ypTis. After completion of NCT, mammographic findings of patients with ypT0 were significantly noted as either complete response or undetected.

**Table 2:** Mammographic findings of patients with ypT0 and ypTis.

Parameters	ypT0 (%)	ypTis (%)	*P*-value
Before NCT			
Mean Size ± SD	29.4 ± 21.8	33.7 ± 21.0	0.287^a^
Extent			
Single	46 (63.0)	27 (37.0)	0.105
Multiple	21 (47.7)	23 (52.3)	
Parenchymal pattern			
b	19 (65.5)	10 (34.5)	0.078^b^
c	41 (51.2)	39 (48.8)	
d	7 (87.5)	1 (12.5)	
Presentation of main lesion			
Mass alone	48 (70.6)	20 (29.4)	<0.001
Microcalcifications ± mass	11 (28.9)	27 (71.1)	
Undetected	8 (72.7)	3 (27.3)	
Associated microcalcifications			
Present	15 (31.2)	33 (68.8)	<0.001
Absent	52 (75.4)	17 (24.6)	
Shape			
Round or oval	38 (63.3)	22 (36.7)	0.252
Irregular	15 (57.7)	11 (42.3)	
Undetected	14 (45.2)	17 (54.8)	
Margin			
Circumscribed	4 (100.0)	0 (0.0)	0.302^b^
Microlobulated	7 (58.3)	5 (41.7)	
Spiculated	12 (57.1)	9 (42.9)	
Indistinct	29 (63.0)	17 (37.0)	
Obscured	1 (33.3)	2 (66.7)	
Undetected	14 (45.2)	17 (54.8)	
Density			
High density	23 (65.7)	12 (34.3)	0.295
Equal density	29 (58.0)	21 (42.0)	
Low density or undetected	15 (46.9)	17 (53.1)	
After NCT			
Complete response or undetected	26 (81.2)	6 (18.8)	0.003^b^
Residual microcalcifications alone	3 (50.0)	3 (50.0)	
Residual mass ± microcalcifications	38 (48.1)	41 (51.9)	

^a^Independent samples *t*-test.

^b^Fisher’s exact test.

Table 3 presents the ultrasound results for the ypT0 and ypTis group. Baseline sonographic size, margin, orientation, and echogenicity did not differ between the ypT0 and ypTis group. Round shape of the main lesion, posterior enhancement, and sonographic absence of calcifications were more frequently observed in patients with ypT0. After NCT, ultrasound findings of patients attaining ypT0 showed a higher proportion of complete response.

**Table 3:** Ultrasound findings of patients with ypT0 and ypTis.

Parameters	ypT0 (%)	ypTis (%)	*P*-value
Before NCT			
Mean Size ± SD	29.9 ± 20.0	31.6 ± 18.5	0.641^a^
Shape			
Oval	37 (58.7)	26 (41.3)	0.015
Round	11 (91.7)	1 (8.3)	
Irregular	19 (45.2)	23 (54.8)	
Margin			
Circumscribed	5 (100.0)	0 (0.0)	0.166
Indistinct	23 (60.5)	15 (39.5)	
Angular	9 (69.2)	4 (30.8)	
Microlobulated	20 (51.3)	19 (48.7)	
Spiculated	10 (45.5)	12 (54.5)	
Orientation			
Parallel	38 (50.7)	37 (49.3)	0.054
Non-parallel	29 (69.0)	13 (31.0)	
Echogenicity			
Hyper-echo	1 (33.3)	2 (66.7)	0.466^b^
Iso-echo	11 (68.8)	5 (31.2)	
Hypo-echo	55 (56.1)	43 (43.9)	
Posterior features			
Enhancement	28 (71.8)	11 (28.2)	0.038
Shadowing	10 (40.0)	15 (60.0)	
No posterior features	29 (54.7)	24 (45.3)	
Calcification			
Present	9 (28.1)	23 (71.9)	<0.001
Absent	58 (68.2)	27 (31.8)	
After NCT			
Complete response	27 (75.0)	9 (25.0)	0.010
Residual disease	40 (49.4)	41 (50.6)	

^a^Independent samples *t*-test.

^b^Fisher’s exact test.

MRI findings are shown in Table 4. Before NCT, the size, shape, and margin of the main lesion, background parenchymal enhancement, internal enhancement, T2WI, presence of necrosis, and peritumoral edema did not differ between the two groups. The main lesion of the ypT0 group was more likely to present as a mass, but non-mass enhancement was more frequent in the ypTis group. MRI findings for patients with ypT0 after NCT mostly indicated complete response.

**Table 4:** Magnetic resonance findings of patients with ypT0 and ypTis.

Parameters	ypT0 (%)	ypTis (%)	*P*-value
Before NCT			
Mean Size ± SD	30.5 ± 19.9	32.1 ± 17.4	0.656^a^
BPE			
1	56 (60.2)	37 (39.8)	0.452^b^
2	8 (44.4)	10 (55.6)	
3	3 (50.0)	3 (50.0)	
Presentation of main lesion			
Mass	59 (62.1)	36 (37.9)	0.028
Non-mass enhancement	8 (36.4)	14 (63.6)	
Shape			
Round	14 (60.9)	9 (39.1)	0.282
Oval	39 (61.9)	24 (38.1)	
Irregular	14 (45.2)	17 (54.8)	
Margin			
Circumscribed	13 (65.0)	7 (35.0)	0.403^b^
Irregular	48 (53.9)	41 (46.1)	
Spiculated	6 (75.0)	2 (25.0)	
Internal enhancement			
Heterogeneous	34 (50.0)	34 (50.0)	0.133
Homogeneous	26 (70.3)	11 (29.7)	
Rim enhancement	7 (58.3)	5 (41.7)	
Time-intensity curve			
Washout	64 (95.5)	49 (98.0)	0.830
Plateau or persistent	3 (4.5)	1 (2.0)	
T2WI			
High	42 (58.3)	30 (41.7)	0.768
Iso or low	25 (55.6)	20 (44.4)	
Presence of necrosis			
No	58 (56.9)	44 (43.1)	0.819
Yes	9 (60.0)	6 (40.0)	
Peritumoral edema			
No	48 (57.1)	36 (42.9)	0.966
Yes	19 (57.6)	14 (42.4)	
After NCT			
Complete response	47 (74.6)	16 (25.4)	<0.001
Residual disease	20 (37.0)	34 (63.0)	

BPE, Background parenchymal enhancement; T2WI, T2-weighted image.

^a^Independent samples *t*-test.

^b^Fisher’s exact test.

**Fig 1:**
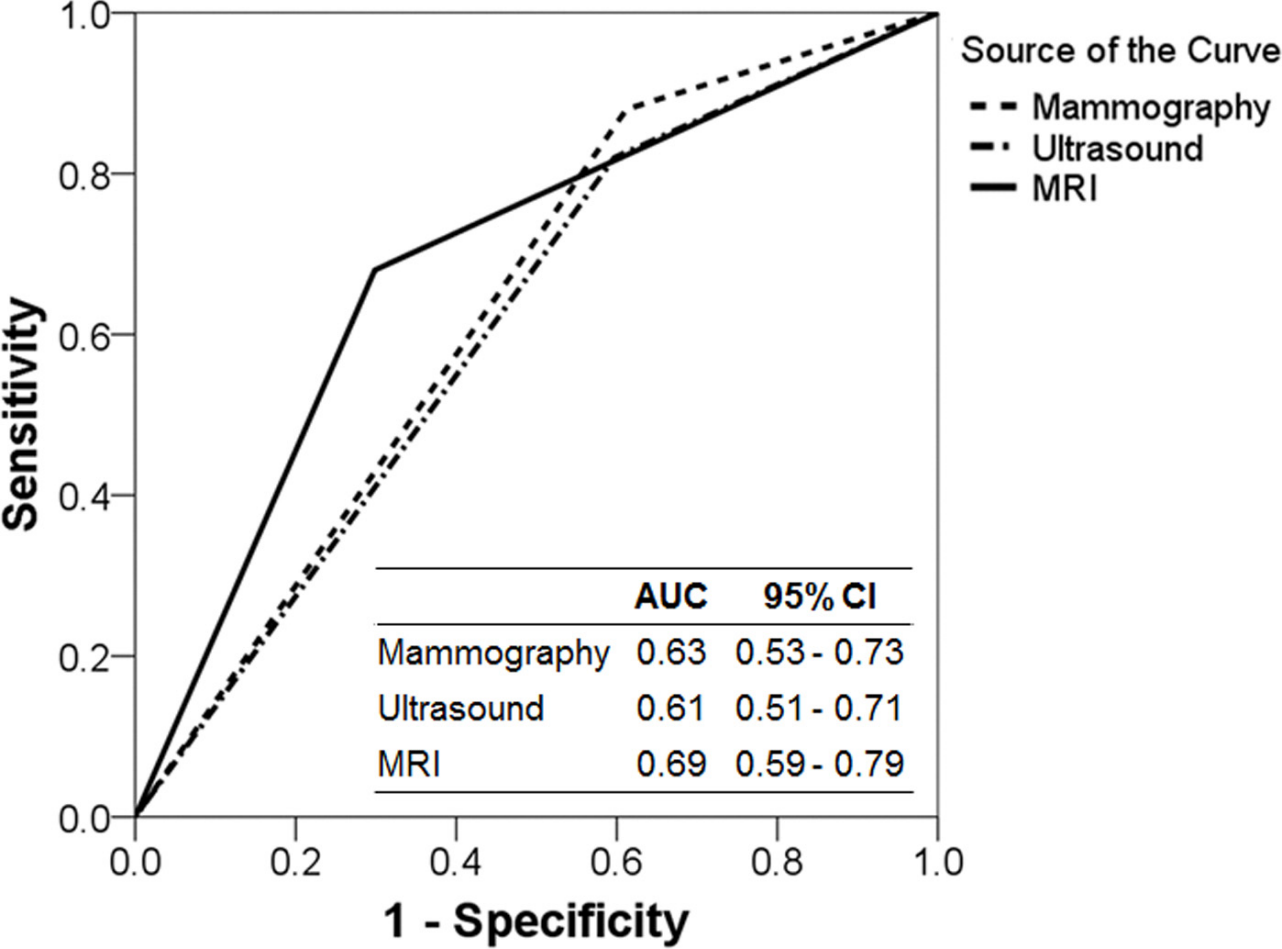
Receiver operating characteristics (ROC) curve analysis for detecting ypTis. AUC, area under the ROC curve; CI, confidence interval; MRI, magnetic resonance imaging.

The sensitivity, specificity, and accuracy for the detection of residual DCIS at the time before surgery was 88.0%, 38.8%, and 59.8% for mammography, respectively, 82.0%, 40.3%, and 58.1% for ultrasound, respectively, and 68.0%, 70.1%, and 62.9% for MRI, respectively. Fig 1 shows ROC curve analysis for detecting ypTis. AUC of mammography, ultrasound, and MRI was 0.63 (95% confidence interval [CI], 0.53–0.73), 0.61 (95% CI, 0.51–0.71), and 0.69 (95% CI, 0.59–0.79), respectively.

Multivariate logistic regression analyses were conducted to identify independent predictors of ypT0 after completion of NCT (Table 5). The triple-negative subtype and complete response in MRI after NCT were significant predictors of ypT0. There was no significant interaction between breast cancer subtypes and MRI results in the multivariate model. Since breast cancer subtypes and MRI findings after NCT were the most important predictors, we conducted stratified analyses according to breast cancer subtype to explore the relationship between MRI findings and residual tumor burden after NCT (Table 6). In all breast cancer subtypes except the HER2-positive subtype, breast MRI well predicted ypT0 with statistical significance. In HRs+/HER2- and HRs+/HER2+ tumors, approximately two-thirds of the patients with complete response observed on MRI after NCT were determined to be ypT0 after surgery, with significant difference. In particular, 22 of 40 patients with HRs-/HER2- tumors showed complete response according to MRI after NCT and among these patients, 21 (95.5%) were ypT0. However, there were no significant differences in patients with HRs-/HER2+ tumors. During the study period, only 2 patients were treated with trastuzumab in combination with chemotherapy since anti-HER2 targeted therapy for neoadjuvant treatment is not covered by the Korean National Health Insurance. When these 2 cases were excluded, MRI findings after NCT were not associated with residual disease in 23 HRs-/HER2+ tumors (*p* = 0.193, Fisher’s exact test).

**Table 5:** Predictors of ypT0 in the breast after completion of NCT.

Parameters	Odds ratio	95% CI	*P*-value
Breast cancer subtype			0.098
HRs+/HER2-	Ref		
HRs+/HER2+	0.70	0.16–3.00	0.633
HRs-/HER2+	0.97	0.26–3.54	0.958
HRs-/HER2-	4.23	1.11–16.09	0.034
MMG presentation of the main lesion			
Mass or undetected	Ref		
Microcalcifications ± mass	0.29	0.03–2.51	0.263
MMG associated microcalcifications			
Absent	Ref		
Present	0.84	0.13–5.60	0.855
US shape			0.370
Irregular	Ref		
Oval	1.11	0.39–3.17	0.846
Round	5.83	0.49–69.80	0.164
US posterior features			0.543
No posterior features	Ref		
Shadowing	1.40	0.38–3.17	0.846
Enhancement	1.92	0.60–6.21	0.275
US calcification			
No	Ref		
Yes	1.34	0.22–8.27	0.751
MRI presentation of the main lesion			
Mass	Ref		
Non-mass enhancement	1.18	0.32–4.31	0.807
MMG after NCT			
Residual disease	Ref		
Complete response	0.90	0.20–4.06	0.890
US after NCT			
Residual disease	Ref		
Complete response	2.69	0.79–9.19	0.114
MRI after NCT			
Residual disease	Ref		
Complete response	5.23	1.53–17.85	0.008

CI, confidence interval; Ref, reference; MMG, mammography; US, ultrasound; MRI, magnetic resonance imaging.

**Table 6:** MRI findings after NCT according to postoperative pathologic results stratified by breast cancer subtype.

MRI after NCT	ypT0	ypTis	*P*-value	*P*-value^a^
HRs+/HER2- (n = 33)				<0.001
Complete response	13 (68.4)	6 (31.6)	0.024	
Residual disease	4 (28.6)	10 (71.4)		
HRs+/HER2+ (n = 19)				
Complete response	6 (66.7)	3 (33.3)	0.020^b^	
Residual disease	1 (10.0)	9 (90.0)		
HRs-/HER2+ (n = 25)				
Complete response	7 (53.8)	6 (46.2)	0.226^b^	
Residual disease	3 (25.0)	9 (75.0)		
HRs-/HER2- (n = 40)				
Complete response	21 (95.5)	1 (4.5)	0.033^b^	
Residual disease	12 (66.7)	6 (33.3)		

^a^Cochran-Mantel-Haenszel test.

^b^Fisher’s exact test.

## Discussion

Recent pooled analyses of clinical trials of NCT indicate that achievement of pCR is associated with improved survival among breast cancer patients [3]. However, the implications of these findings are thought to vary among breast cancer subtypes [12]. Although several classifications have been suggested for pathologic response to NCT in the breast, pCR of the breast in practice is defined as no residual carcinoma (ypT0) or no residual invasive tumor with DCIS present (ypTis) [13,14]. The prognostic implications of pCR are somewhat controversial but in general, there are no differences in survival between patients with ypT0 and patients with ypT0/is when ypN0 is attained [3,12]. However, ypT0 and ypTis cannot be accurately distinguished before definitive surgery, and some tumors do not respond in uniform patterns to NCT [15,16]. The major clinical advantage of NCT is an increased success rate of breast-conservation surgery, which can be applied to patients with favorable response to NCT who fulfill the criteria for breast-conservation surgery. Therefore, monitoring response to NCT and evaluating residual tumor extent before surgery are clinically important practices.

Mammography is clinically useful for evaluating the extent of malignant-appearing calcifications. Lesions with residual DCIS frequently show calcifications on pre-chemotherapy mammograms [6], as observed in this study. When main lesions initially presented as microcalcifications with or without masses or when associated microcalcifications were detected, ypTis was more likely to be observed after NCT. However, calcifications are also indicative of necrotic tumor cells in patients who have received NCT. Our results showed that approximately half of all cases with residual masses or microcalcifications after completion of NCT were identified as ypT0. This result is similar to previous studies which indicated that remnant calcifications after NCT are not correlated with residual tumor burden [6,17,18]. Moreover, 33.3% of stable microcalcifications and 27.7% of newly developed or additional calcifications after NCT turned out to be pCR at the time of surgery, while 100% of calcifications in cases with increased mass showed residual malignancy [19]. In HER2-positive breast cancers, adjacent DCIS could be completely eradicated by NCT combined with trastuzumab [20]. Therefore, remnant calcifications on mammography after NCT should not be considered to constitute evidence of residual DCIS. While practical guidelines indicate that findings of diffuse suspicious or malignant-appearing microcalcifications absolutely contraindicate breast-conserving therapy [21], a comprehensive clinical and imaging analysis which considers breast cancer subtypes and therapeutic regimens is necessary to plan surgery after completion of NCT.

Recently, Lee et al. [22] summarized inaccuracies among current practical tools used to evaluate residual tumor volumes in response to NCT and demonstrated that two-dimensional and three-dimensional ultrasound and breast MRI show similar performances for the estimation of residual breast cancer volume and prediction of pCR. In a retrospective analysis of patients enrolled in the GeparTrio trial, ultrasound showed a high sensitivity for predicting ypT0 and ypN0 and modestly improved the prediction of pCR by patient characteristics, which was concluded to be a potentially useful modality for early prediction of pCR, despite breast MRI not being included in the study [23]. In addition, ultrasound provides clinical advantages over MRI including lower complexity, easier accessibility, shorter procedure time, easier interpretation, cheaper costs, and lack of the hazards associated with contrast agents [22,23]. In the present study, univariate analyses demonstrated that round shape on baseline sonographic analyses, posterior features, sonographic absence of calcification, and complete response after NCT were associated with a higher possibility of ypT0. However, there was no significant effect observed in multivariate analysis, and more studies are required to confirm the role of ultrasound in the prediction of residual tumor burden after NCT. Some potential explanations could lie in the fact that all cases included in our study showed pCR at the time of surgery, which means that residual disease was determined by *in situ* components of permanent pathology. No imaging modality other than mammography is currently accepted in the evaluation of DCIS. For example, ultrasound has limited ability to detect microcalcifications due to technical issues [24]. Although there are several circumstances in which ultrasound may be beneficial in the evaluation of patients with DCIS, sonographic findings of DCIS are very subtle. Therefore, even though ultrasound might help predict pCR based [23], its ability to differentiate ypT0 from ypTis needs to be investigated. In addition, interobserver variability is a well-known limitation of ultrasound [24]. Examining the exact primary site can be difficult in cases with markedly decreased tumor burden size due to the limited number of landmarks available for ultrasound.

A meta-analysis of MRI in the prediction of pCR after NCT revealed a high specificity of 0.91 and a relatively low sensitivity of 0.63 [4]. However, the performance of MRI can be influenced by pCR rates, Ki-67 index, and breast cancer subtype [4,8,25,26]. The accuracy of MRI for predicting residual tumor size was greatest in patients with the triple-negative phenotype or HER2-positive breast cancers, and a better correlation was noted in the triple-negative subtype with higher Ki-67 levels [8,25,26]. In this study, the triple-negative breast cancer subtype and complete response on MRI after NCT were independent predictors for discriminating ypT0 from ypTis. This is supported by previous study results which have shown that mass enhancement is an imaging characteristic of triple-negative breast cancer and that associated DCIS is rare in cases without non-mass enhancement [27]. However, Moon et al. [7] reported that the use of HER2-targeted agents resulted in less accurate MRI in patients with HER2-positive tumors. In the present study, although most patients with HER2-positive tumors did not receive HER2-directed therapy, MRI after NCT showed poor performance for the prediction of ypT0 in HRs-/HER2+ tumors. Breast cancer subtypes are associated with pCR rates after NCT, and the incorporation of HER2-targeted agents into NCT significantly improved pCR rates in HER2-positive breast cancers [2,12,28]. The relationship between biologic mechanisms and MRI used to discriminate ypT0 from ypTis in HER2-positive tumors has yet to be determined.

Potential limitations of the present study are that it was a retrospective analysis using a single institution database, and that the interpretations of imaging modalities were performed by a single radiologist (although the radiologist was blinded to clinicopathological information). In addition, patients with non-pCR after NCT were not analyzed, and confirmative parameters for the discrimination of ypT0 from ypTis or residual invasive carcinoma were not evaluated. Nevertheless, our study has two major strengths. One is that more than 100 cases attaining pCR in the breast after NCT were investigated and the other is that all patients underwent mammography, ultrasound, and breast MRI prior to and after NCT. Therefore, we were able to comprehensively analyze the impact of all three imaging modalities before and after NCT on the prediction of pCR while considering clinicopathological factors including breast cancer subtype. Our multivariate analyses suggest that MRI after NCT affects discrimination between ypT0 and ypTis differently according to breast tumor phenotype. Of note, since the high false-positive rate and the subsequently frequent overcall rate are weaknesses of MRI, further study with a larger multicenter cohort is necessary to validate our results and to evaluate the clinical benefits and risks of MRI.

In conclusion, we demonstrated that the triple-negative breast cancer subtype and complete response in MRI after NCT are independent predictors of ypT0. Among imaging modalities, breast MRI could be suggested as a modality that accurately discriminates between ypT0 and ypTis after NCT, especially in patients with triple-negative breast cancer. However, statistically low AUC value and relatively high false-positive rate of MRI given in the present study suggest that our findings are not definitive and additional study should be conducted. Until finding out more clinically relevant imaging modalities and appropriate patient selection criteria, this information can be useful in the evaluation of tumor response to NCT and in the planning of surgery for breast cancer patients of all subtypes except for HER2-positive tumors after NCT.

## Supporting Information

S1 Appendix The raw data of image review. (PDF)
