# Supplementary material for: Magnetic Resonance Imaging after Completion of Neoadjuvant Chemotherapy Can Accurately Discriminate between No Residual Carcinoma and Residual Ductal Carcinoma In Situ in Patients with Triple-Negative Breast Cancer
Source: PLoS One. 2016 Feb 11;11(2):e0149347. doi: 10.1371/journal.pone.0149347 (PMC4750980; doi:10.1371/journal.pone.0149347)
Supplement: S1 Appendix — (PDF) [file pone.0149347.s001.pdf]

SN	T	T_multi	T_ROC	MMG_size	MMG_exte	MMG_patt	MMG_mai
7081	1	1	0	15	1	3	0
7118	1	1	0	50	1	3	1
7148	2	0	1	20	1	2	0
7194	2	0	1	35	1	3	0
7297	1	1	0	0	1	3	2
7346	1	1	0	25	2	2	1
7393	1	1	0	24	1	3	0
7437	2	0	1	21	2	3	1
7477	1	1	0	58	1	2	0
7517	1	1	0	0	2	2	2
7628	1	1	0	0	1	3	2
7666	2	0	1	16	1	3	1
7669	1	1	0	58	1	3	0
7744	1	1	0	29	1	3	0
7795	1	1	0	22	1	2	0
7807	1	1	0	20	1	3	0
7811	2	0	1	17	1	3	0
7965	2	0	1	45	1	3	1
8012	2	0	1	29	1	3	1
8031	2	0	1	80	2	3	0
8107	2	0	1	47	2	3	0
8126	1	1	0	21	1	3	1
8137	2	0	1	21	2	3	1
8144	2	0	1	22	2	4	0
8178	1	1	0	65	2	4	1
8190	1	1	0	47	1	2	0
8198	1	1	0	36	2	3	0
8230	1	1	0	45	1	2	0
8236	1	1	0	15	1	2	0
8238	2	0	1	16	2	3	2
8240	1	1	0	52	2	3	0
8241	1	1	0	19	1	2	0
8243	2	0	1	52	2	3	1
8280	2	0	1	40	2	2	1
8299	1	1	0	14	1	3	0
8310	1	1	0	55	1	3	0
8312	1	1	0	17	1	3	0
8330	2	0	1	20	2	3	0
8408	1	1	0	43	1	3	0
8409	2	0	1	26	2	3	0
8417	1	1	0	26	1	3	0
8459	2	0	1	19	1	2	0

8484	1	1	0	24	1	3	0
8495	1	1	0	15	1	3	0
8496	1	1	0	81	2	3	0
8503	2	0	1	52	2	3	1
8513	2	0	1	43	2	3	1
8521	2	0	1	34	1	3	0
8550	2	0	1	20	1	3	0
8574	1	1	0	12	1	2	0
8580	1	1	0	36	1	2	1
8616	2	0	1	21	2	2	0
8622	1	1	0	0	2	4	2
8653	2	0	1	27	1	3	0
8680	1	1	0	77	2	4	1
8724	2	0	1	65	1	3	1
8767	2	0	1	10	1	3	1
8773	1	1	0	20	1	3	1
8784	1	1	0	17	2	2	0
8793	1	1	0	35	2	3	0
8838	2	0	1	20	1	3	1
8878	1	1	0	10	1	3	0
8879	2	0	1	27	1	3	0
8904	2	0	1	40	1	3	1
8942	1	1	0	0	1	3	2
9000	2	0	1	32	2	3	1
9055	1	1	0	24	1	3	0
9063	1	1	0	20	1	3	0
9064	2	0	1	60	1	3	1
9068	1	1	0	45	2	3	1
9113	2	0	1	19	1	2	1
9148	1	1	0	52	2	3	1
9165	1	1	0	30	1	3	0
9166	1	1	0	27	2	3	0
9228	2	0	1	9	1	2	1
9263	1	1	0	20	1	3	0
9277	1	1	0	24	1	3	0
9287	1	1	0	24	2	2	0
9296	2	0	1	28	2	3	0
9323	2	0	1	40	1	3	1
9337	2	0	1	36	2	3	1
9352	1	1	0	24	1	2	0
9357	1	1	0	78	2	2	0
9375	1	1	0	14	1	3	1
9385	2	0	1	22	1	2	0

9395	2	0	1	65	2	2	1
9406	2	0	1	31	1	3	1
9421	2	0	1	0	2	3	2
9487	2	0	1	22	1	3	0
9535	2	0	1	29	1	2	0
9598	1	1	0	10	1	2	0
9616	1	1	0	0	1	3	2
9675	1	1	0	30	2	2	0
9777	2	0	1	30	1	3	1
9796	2	0	1	27	1	2	1
9827	1	1	0	38	1	4	0
9860	1	1	0	0	1	4	2
9900	2	0	1	110	2	3	1
9903	1	1	0	82	2	3	1
969300	1	1	0	34	1	3	0
969400	1	1	0	14	2	3	0
970500	2	0	1	0	1	3	2
972600	1	1	0	50	2	3	0
973900	1	1	0	22	1	3	0
974400	1	1	0	24	1	3	0
974500	2	0	1	16	1	3	0
975300	2	0	1	50	2	3	1
977100	2	0	1	40	2	3	1
977200	1	1	0	16	1	2	0
981200	1	1	0	20	1	2	0
982100	1	1	0	90	2	2	0
982800	2	0	1	76	2	3	1
984600	1	1	0	28	1	3	0
985700	1	1	0	19	1	3	0
987400	1	1	0	30	1	4	0
991100	2	0	1	59	2	3	0
992500	1	1	0	0	2	4	2

MMG_assc	MMG_shap	MMG_den:	US_size	US_shape	US_margin	US_orienta	US_orienta
0	1	iso	15	1	circumscrik	not paralle	1
1	1	high	44	1	indistinct	parallel	0
0	1	iso	11	1	indistinct	not paralle	1
0	1	high	23	1	microlobul	parallel	0
0	0	no	13	1	spiculated	parallel	0
1	2	iso	19	3	spiculated	not paralle	1
1	1	iso	24	1	indistinct	not paralle	1
1	2	iso	25	3	indistinct	not paralle	1
0	1	iso	49	1	indistinct	parallel	0
0	0	no	4	1	indistinct	parallel	0
0	0	no	15	1	indistinct	parallel	0
1	2	iso	13	3	microlobul	parallel	0
0	1	high	53	2	circumscrik	parallel	0
0	1	iso	24	1	microlobul	parallel	0
0	1	high	19	2	spiculated	not paralle	1
1	1	high	22	1	angular	parallel	0
1	1	iso	20	3	microlobul	parallel	0
1	1	iso	42	3	microlobul	parallel	0
1	1	iso	27	1	microlobul	parallel	0
0	2	iso	80	1	spiculated	parallel	0
0	2	high	16	3	spiculated	not paralle	1
1	2	iso	23	3	angular	not paralle	1
1	1	iso	22	3	spiculated	not paralle	1
1	1	iso	43	1	indistinct	parallel	0
1	0	no	50	1	indistinct	parallel	0
0	2	high	26	1	microlobul	not paralle	1
0	1	high	36	1	indistinct	parallel	0
0	1	iso	49	1	circumscrik	parallel	0
0	1	iso	16	1	indistinct	not paralle	1
1	0	no	33	1	indistinct	parallel	0
1	1	high	48	1	angular	parallel	0
0	1	high	19	2	microlobul	not paralle	1
1	0	no	60	3	spiculated	not paralle	1
1	0	no	40	3	angular	parallel	0
0	1	iso	11	1	circumscrik	parallel	0
0	1	high	52	2	circumscrik	not paralle	1
0	1	high	14	2	microlobul	not paralle	1
1	2	high	21	1	microlobul	not paralle	1
0	1	high	32	2	microlobul	not paralle	1
0	1	high	19	1	microlobul	not paralle	1
0	1	high	31	3	indistinct	parallel	0
1	1	high	22	1	microlobul	parallel	0

1 1	iso	13	2	indistinct	not paralle	1
0 1	iso	17	3	spiculated	not paralle	1
0 2	high	80	1	microlobul	parallel	0
1 0	no	55	1	indistinct	parallel	0
1 0	no	50	1	indistinct	not paralle	1
0 1	high	31	3	microlobul	parallel	0
0 1	iso	27	1	indistinct	not paralle	1
0 1	iso	9	1	angular	parallel	0
1 2	high	22	1	indistinct	not paralle	1
0 1	iso	31	1	indistinct	parallel	0
0 0	no	30	1	indistinct	parallel	0
0 1	high	27	1	microlobul	parallel	0
1 0	no	70	1	indistinct	parallel	0
1 0	no	18	1	microlobul	parallel	0
1 0	no	15	1	indistinct	parallel	0
1 0	no	11	1	indistinct	not paralle	1
0 1	iso	13	1	indistinct	not paralle	1
0 2	iso	32	3	spiculated	not paralle	1
1 1	iso	22	1	microlobul	parallel	0
0 1	iso	9	1	indistinct	parallel	0
0 1	iso	20	3	indistinct	parallel	0
1 0	no	40	1	indistinct	parallel	0
0 0	no	14	1	indistinct	parallel	0
1 0	no	39	1	microlobul	parallel	0
0 1	high	27	1	microlobul	not paralle	1
0 1	high	16	1	indistinct	not paralle	1
1 1	high	51	3	microlobul	parallel	0
1 1	high	38	1	indistinct	parallel	0
1 0	no	25	1	spiculated	parallel	0
1 0	no	49	1	indistinct	parallel	0
0 1	iso	25	2	microlobul	not paralle	1
0 1	iso	30	1	microlobul	not paralle	1
1 1	iso	10	3	indistinct	not paralle	1
0 2	iso	22	3	microlobul	not paralle	1
0 1	iso	17	2	indistinct	not paralle	1
0 2	high	18	3	spiculated	parallel	0
1 2	high	43	1	spiculated	parallel	0
1 0	no	20	3	indistinct	parallel	0
1 0	no	20	1	spiculated	parallel	0
0 1	iso	23	1	microlobul	parallel	0
0 2	high	80	3	spiculated	parallel	0
1 0	no	10	1	indistinct	parallel	0
0 1	high	23	1	microlobul	parallel	0

1	2	iso	60	3	indistinct	not paralle	1
1	1	iso	25	1	angular	parallel	0
0	0	no	15	3	spiculated	parallel	0
0	2	high	22	1	microlobul	parallel	0
0	1	high	24	3	spiculated	not paralle	1
0	2	high	10	2	microlobul	not paralle	1
0	0	no	27	3	angular	parallel	0
0	2	iso	44	3	microlobul	parallel	0
1	2	iso	22	3	angular	parallel	0
1	2	iso	25	3	spiculated	parallel	0
0	1	iso	38	1	spiculated	parallel	0
0	0	no	27	3	angular	parallel	0
1	0	no	100	3	microlobul	parallel	0
1	0	no	100	3	microlobul	parallel	0
0	2	high	23	1	angular	not paralle	1
0	1	iso	15	3	microlobul	not paralle	1
0	0	no	11	2	microlobul	parallel	0
0	2	iso	50	1	microlobul	parallel	0
0	1	iso	21	1	microlobul	parallel	0
0	1	iso	14	1	microlobul	parallel	0
0	1	iso	22	3	spiculated	parallel	0
1	1	iso	26	1	indistinct	parallel	0
1	0	no	26	3	angular	parallel	0
0	1	high	17	2	spiculated	parallel	0
0	1	iso	24	3	angular	not paralle	1
0	2	high	90	3	indistinct	not paralle	1
1	0	no	69	3	spiculated	not paralle	1
0	2	iso	28	3	angular	parallel	0
0	1	no	20	3	spiculated	parallel	0
0	1	iso	37	3	microlobul	not paralle	1
0	2	iso	50	3	microlobul	parallel	0
0	0	no	37	3	microlobul	parallel	0

US_echoge	US_posteri	US_posteri	US_calcific	MR_size	MR_BPE	MR_preser	MR_shape
hypo	enhancem	2	0	20	2	mass	oval
hypo	enhancem	2	0	37	3	mass	oval
hypo	shadowing	1	0	20	1	mass	irregular
hypo	enhancem	2	0	30	2	mass	oval
hypo	no	0	0	10	2	mass	round
iso	shadowing	1	0	22	1	mass	irregular
hypo	enhancem	2	1	16	1	mass	round
hypo	no	0	1	29	1	mass	irregular
hypo	enhancem	2	0	67	1	non-mass	oval
iso	no	0	0	4	1	mass	round
iso	enhancem	2	0	19	1	mass	oval
hypo	shadowing	1	1	28	2	mass	irregular
hypo	enhancem	2	0	47	1	mass	oval
hypo	no	0	0	29	1	mass	oval
hypo	shadowing	1	0	20	1	mass	oval
hypo	enhancem	2	0	20	1	mass	oval
hypo	enhancem	2	0	22	2	mass	oval
hypo	shadowing	1	1	37	1	mass	oval
hypo	no	0	1	30	3	mass	oval
hypo	shadowing	1	0	78	3	non-mass	irregular
hypo	no	0	0	36	2	non-mass	irregular
iso	enhancem	2	1	18	2	mass	oval
hypo	no	0	1	18	1	mass	round
hypo	no	0	1	39	1	mass	oval
hypo	shadowing	1	1	63	1	mass	irregular
hypo	no	0	0	34	1	mass	round
hypo	no	0	0	39	1	non-mass	oval
hypo	enhancem	2	0	55	1	mass	oval
hypo	enhancem	2	0	13	1	mass	round
hypo	shadowing	1	0	29	1	mass	oval
hypo	shadowing	1	0	40	1	mass	round
iso	enhancem	2	0	23	1	mass	round
hypo	shadowing	1	0	60	2	non-mass	irregular
hypo	shadowing	1	0	43	1	mass	irregular
hypo	no	0	0	9	1	mass	oval
hypo	shadowing	1	0	55	2	mass	round
hypo	enhancem	2	0	14	1	mass	round
hypo	enhancem	2	0	28	2	mass	irregular
hypo	enhancem	2	0	30	1	mass	round
hypo	enhancem	2	0	22	1	mass	round
hypo	no	0	0	26	3	mass	oval
hypo	no	0	0	18	1	mass	oval

hypo	no	0	0	20	1	mass	round
hypo	no	0	0	15	1	mass	oval
hyper	shadowing	1	0	71	1	mass	irregular
hyper	no	0	0	33	2	non-mass	oval
hypo	shadowing	1	1	40	1	mass	oval
hypo	enhancem	2	0	30	1	mass	oval
hypo	enhancem	2	0	25	3	mass	oval
iso	no	0	0	9	1	mass	oval
hypo	no	0	1	28	1	mass	oval
hypo	no	0	0	29	1	mass	oval
hypo	no	0	0	32	1	mass	oval
hypo	enhancem	2	0	29	1	mass	round
hypo	no	0	1	65	1	non-mass	irregular
hypo	no	0	0	18	1	mass	oval
hypo	no	0	1	12	1	non-mass	oval
iso	no	0	1	24	1	non-mass	oval
hypo	enhancem	2	0	16	1	mass	oval
hypo	no	0	0	28	1	mass	oval
hypo	enhancem	2	1	21	1	mass	oval
hypo	no	0	0	10	1	mass	oval
hypo	no	0	0	20	1	non-mass	oval
iso	shadowing	1	1	45	1	mass	oval
iso	no	0	0	13	1	mass	oval
hypo	no	0	1	32	1	mass	oval
hypo	no	0	0	24	1	mass	oval
hypo	enhancem	2	0	22	1	mass	oval
hypo	no	0	1	51	1	non-mass	oval
hypo	enhancem	2	0	38	1	mass	oval
hypo	enhancem	2	1	18	1	mass	oval
hypo	no	0	1	42	1	mass	oval
hypo	enhancem	2	0	24	2	mass	oval
hypo	enhancem	2	0	28	1	mass	oval
hypo	shadowing	1	1	14	1	mass	irregular
hypo	enhancem	2	0	27	2	mass	irregular
hypo	no	0	0	24	1	mass	oval
iso	no	0	0	17	1	mass	irregular
hypo	shadowing	1	0	43	1	mass	oval
hyper	no	0	1	47	1	non-mass	irregular
hypo	no	0	1	30	1	non-mass	oval
hypo	enhancem	2	0	20	1	mass	oval
iso	shadowing	1	0	70	1	non-mass	irregular
hypo	no	0	1	12	1	mass	oval
hypo	enhancem	2	0	24	1	mass	oval

hypo	shadowing	1	1	65	1	non-mass	irregular
hypo	enhancem	2	1	34	1	mass	round
iso	shadowing	1	0	8	1	mass	round
hypo	no	0	0	19	2	mass	oval
hypo	no	0	0	29	2	mass	irregular
hypo	no	0	0	20	1	mass	oval
hypo	enhancem	2	0	15	1	mass	irregular
hypo	no	0	0	37	1	mass	irregular
hypo	shadowing	1	0	27	1	non-mass	irregular
iso	no	0	1	27	1	mass	irregular
hypo	enhancem	2	0	26	1	mass	round
iso	enhancem	2	0	28	3	mass	round
hypo	shadowing	1	1	93	1	non-mass	irregular
hypo	shadowing	1	1	95	2	non-mass	irregular
hypo	enhancem	2	0	23	1	mass	oval
hypo	no	0	0	13	1	mass	round
hypo	no	0	0	11	2	mass	round
hypo	shadowing	1	0	53	1	non-mass	irregular
hypo	enhancem	2	0	22	1	mass	oval
hypo	no	0	0	16	1	mass	oval
iso	no	0	0	17	1	mass	round
iso	no	0	1	14	1	mass	round
hypo	no	0	1	16	1	mass	round
hypo	no	0	0	19	1	mass	oval
hypo	enhancem	2	0	19	1	mass	oval
hypo	shadowing	1	0	100	1	non-mass	irregular
hypo	no	0	1	68	1	non-mass	irregular
hypo	enhancem	2	0	26	1	mass	oval
hypo	no	0	0	24	1	mass	irregular
hypo	enhancem	2	0	57	1	mass	irregular
hypo	no	0	0	49	1	non-mass	irregular
hypo	no	0	0	43	2	mass	oval

MR_margi	MR_enh_p	MR_T2WI	MR_Delay	MR_necros	MR_peritu	MMG_redi	MMG_resi
spiculated	rim	high	wash	no	no	0	0
irregular	rim	high	wash	yes	no	2	1
irregular	homo	high	wash	no	no	2	1
irregular	hetero	high	wash	no	no	2	1
spiculated	homo	iso	wash	no	no	0	0
spiculated	rim	high	wash	no	no	2	1
irregular	homo	high	wash	no	no	2	1
irregular	homo	high	wash	no	no	2	1
irregular	rim	high	wash	yes	no	2	1
well	homo	iso	wash	no	no	0	0
irregular	rim	high	wash	yes	no	0	0
spiculated	hetero	iso	wash	no	no	2	1
well	homo	high	wash	no	yes	2	1
irregular	hetero	iso	wash	no	no	2	1
well	homo	iso	wash	no	no	2	1
well	homo	high	wash	no	no	2	1
irregular	hetero	high	wash	no	no	2	1
irregular	hetero	high	wash	no	no	2	1
irregular	rim	high	wash	yes	yes	2	1
irregular	hetero	high	wash	yes	yes	2	1
irregular	hetero	iso	wash	no	no	2	1
irregular	homo	iso	wash	no	yes	2	1
irregular	hetero	high	wash	no	no	2	1
spiculated	hetero	iso	wash	no	no	2	1
irregular	hetero	iso	wash	no	yes	2	1
irregular	homo	high	wash	no	yes	2	1
irregular	homo	iso	wash	no	yes	2	1
irregular	hetero	high	wash	yes	yes	2	1
well	homo	high	wash	no	yes	0	0
irregular	hetero	high	wash	no	yes	2	1
irregular	homo	high	wash	no	yes	2	1
irregular	hetero	high	wash	no	yes	2	1
irregular	hetero	iso	wash	no	yes	2	1
irregular	hetero	iso	wash	no	yes	2	1
irregular	homo	high	wash	no	no	0	0
well	hetero	iso	wash	no	yes	2	1
irregular	homo	high	wash	no	no	2	1
irregular	homo	high	wash	no	no	2	1
well	rim	high	wash	yes	no	2	1
well	rim	high	wash	yes	yes	0	0
irregular	hetero	high	wash	no	yes	0	0
well	hetero	high	wash	no	yes	2	1

irregular	hetero	high	wash	no	no	2	1
spiculated	homo	high	wash	no	no	0	0
irregular	hetero	iso	wash	no	yes	0	0
irregular	rim	high	plateau	no	no	2	1
irregular	hetero	iso	wash	no	yes	2	1
well	hetero	high	wash	no	no	2	1
well	homo	high	wash	no	no	0	0
well	homo	iso	plateau	no	no	2	1
irregular	homo	high	wash	no	no	2	1
irregular	hetero	high	wash	no	yes	0	0
irregular	hetero	iso	wash	no	no	0	0
irregular	hetero	high	wash	no	yes	2	1
irregular	hetero	iso	wash	no	no	2	1
irregular	homo	iso	wash	no	no	2	1
irregular	hetero	iso	wash	no	no	2	1
irregular	hetero	iso	plateau	no	no	2	1
irregular	hetero	high	wash	no	yes	0	0
irregular	hetero	high	plateau	no	no	2	1
irregular	hetero	high	wash	no	no	2	1
irregular	homo	high	wash	no	no	0	0
irregular	homo	high	wash	no	no	2	1
well	hetero	high	wash	no	yes	2	1
irregular	homo	high	wash	no	no	0	0
irregular	hetero	high	wash	no	yes	2	1
irregular	hetero	high	wash	no	yes	2	1
well	hetero	high	wash	no	yes	0	0
irregular	hetero	iso	wash	no	yes	2	1
irregular	hetero	high	wash	no	no	2	1
irregular	hetero	high	wash	no	no	2	1
irregular	hetero	high	wash	no	yes	2	1
well	rim	high	wash	no	no	0	0
well	homo	high	wash	no	no	0	0
well	hetero	high	wash	no	no	2	1
spiculated	homo	high	wash	no	no	0	0
irregular	hetero	high	wash	no	no	2	1
irregular	hetero	iso	wash	no	no	0	0
irregular	hetero	high	wash	no	no	2	1
irregular	hetero	high	wash	no	no	2	1
irregular	homo	high	wash	no	no	2	1
irregular	homo	iso	wash	no	no	2	1
irregular	homo	iso	wash	no	yes	2	1
irregular	hetero	iso	wash	no	no	1	1
irregular	hetero	iso	wash	yes	no	0	0

irregular	hetero	high	wash	no	yes	1	1
irregular	hetero	iso	wash	yes	no	2	1
well	homo	iso	wash	no	no	2	1
irregular	hetero	iso	wash	no	no	2	1
irregular	rim	iso	wash	yes	no	2	1
irregular	hetero	high	wash	no	no	0	0
irregular	hetero	high	wash	yes	no	2	1
irregular	hetero	high	wash	no	no	0	0
irregular	hetero	high	wash	no	no	2	1
irregular	hetero	high	wash	no	no	1	1
well	hetero	high	wash	no	no	0	0
irregular	hetero	iso	wash	no	no	2	1
irregular	rim	iso	wash	no	no	2	1
irregular	hetero	iso	wash	no	no	2	1
irregular	homo	high	wash	yes	no	2	1
irregular	hetero	high	wash	no	no	0	0
irregular	hetero	iso	wash	no	no	0	0
irregular	hetero	iso	wash	no	no	2	1
irregular	hetero	iso	wash	yes	no	1	1
irregular	hetero	high	wash	no	no	0	0
irregular	homo	iso	wash	no	no	0	0
irregular	homo	iso	wash	no	no	1	1
irregular	hetero	iso	wash	no	no	2	1
irregular	hetero	high	wash	no	no	0	0
well	homo	high	wash	no	no	0	0
irregular	homo	iso	wash	no	yes	2	1
irregular	hetero	high	wash	no	no	2	1
irregular	hetero	iso	wash	no	no	2	1
spiculated	homo	iso	wash	no	no	1	1
irregular	hetero	high	wash	yes	yes	2	1
irregular	homo	iso	wash	no	no	2	1
irregular	hetero	iso	wash	no	no	0	0

US_residua	MR_residu	Age	Age_40Grc	Final_Menc	BMI_Grour	NCT_regim	PreopCTx_
0	0	37	0	0	1	2	AC+Taxote
0	1	27	0	0	0	1	AC+ Taxot
1	1	50	1	1	0	2	AC+Taxote
1	1	42	1	0	0	2	AC+Taxote
1	0	50	1	0	0	1	AC+Taxote
1	1	60	1	1	0	2	AC+Taxote
1	1	50	1	0	0	2	AC+Taxote
1	1	47	1	1	1	1	AC+Taxote
1	1	47	1	0	0	2	AC+Taxote
0	0	54	1	0	1	1	AC+ Taxot
0	1	41	1	0	0	3	taxol+carb
0	1	51	1	0	0	1	AC+Taxote
1	0	46	1	0	1	1	AC+Taxote
1	0	46	1	0	0	1	AC+Taxote
1	0	65	1	1	1	1	AC+Taxote
0	0	56	1	1	0	1	AC+Taxote
1	1	45	1	0	0	1	AC+Taxote
1	1	42	1	0	0	2	AC+Taxote
1	1	48	1	0	0	1	AC+Taxote
1	1	41	1	0	0	2	AC+Taxote
1	1	38	0	0	1	3	NEAT:carb
1	0	47	1	0	0	1	AC+Taxote
1	1	40	0	0	0	1	AC+Taxote
1	1	28	0	0	0	1	AC+Taxote
1	0	55	1	1	0	1	AC+Taxote
1	1	61	1	1	1	3	NEAT:carb
0	0	39	0	0	0	2	AC+Taxote
1	1	71	1	1	1	3	NEAT/CAR
1	0	55	1	1	0	1	AC+Taxote
1	1	67	1	1	0	1	AC+Taxote
1	1	54	1	1	0	3	NEAT: carb
0	1	53	1	1	1	1	AC+Taxote
0	1	45	1	0	1	1	AC+Taxote
1	1	54	1	1	1	1	AC+Taxote
0	0	54	1	1	0	3	HP
1	1	34	0	0	0	3	NEAT:carb
1	1	47	1	0	0	3	neat:carbo
1	1	41	1	0	0	1	AC+Taxote
1	1	38	0	0	0	3	NEAT: Tax
1	0	58	1	1	1	2	AC+Taxote
0	0	44	1	0	1	1	AC+Taxote
1	1	64	1	1	0	1	AC+Taxote

1	1	41	1	0	1	2 AC+Taxote
0	0	55	1	1	0	3 NEAT: Carl
0	0	48	1	1	0	1 AC+Taxote
0	1	50	1	0	0	1 AC+Taxote
1	0	53	1	1	0	1 AC+Taxote
1	1	57	1	1	1	1 AC+Taxote
1	0	52	1	0	0	1 AC+Mono
1	0	67	1	1	1	1 AC+Taxote
0	0	67	1	1	1	1 AC+Taxote
1	0	56	1	1	1	1 AC+ Taxot
0	0	48	1	0	0	1 AC+Mono
1	1	41	1	0	0	1 AC+Mono
0	1	48	1	1	0	1 AC+Mono
0	0	42	1	0	1	2 AC + Taxo
1	0	40	0	0	0	1 AC+Taxote
1	0	46	1	1	0	1 AC+mono
1	0	66	1	1	1	1 AC+Mono
1	0	51	1	0	0	1 AC+Mono
1	1	51	1	1	0	1 AC+Taxote
1	0	66	1	1	0	1 AC+Taxote
1	1	43	1	0	0	1 AC+Taxote
1	1	55	1	1	1	1 AC+ Taxot
1	0	37	0	0	1	1 AC+Taxote
0	1	54	1	1	0	1 AC+Taxote
1	0	57	1	1	1	1 AC+Taxote
0	0	31	0	0	0	3 TAC
1	1	59	1	1	1	1 AC+TAXOT
1	0	43	1	0	0	1 AC+Mono
1	1	63	1	1	0	3 Taxol/Carb
1	0	58	1	1	0	1 AC+Taxote
0	0	47	1	0	1	1 AC+Taxote
0	0	54	1	1	0	1 AC+Taxote
1	1	70	1	1	1	1 AC+Taxote
0	0	34	0	0	1	1 AC+Taxote
0	0	31	0	0	1	1 AC+Taxote
1	0	68	1	1	0	1 AC+Taxote
1	1	58	1	1	0	1 AC+Taxote
1	0	45	1	0	0	1 AC+Taxote
1	0	56	1	1	0	1 AC+Taxote
1	0	38	0	0	1	1 AC 4회, te
1	1	43	1	0	0	1 AC-Taxote
0	0	45	1	0	0	1 Ac+Taxote
1	0	58	1	1	0	1 AC+mono

0	0	59	1	1	0	1	AC+monot
1	1	55	1	1	1	1	4AC-4T
0	0	42	1	0	1	1	AC-Taxote
1	0	37	0	0	0	1	AC-Taxote
1	1	44	1	0	1	1	AC-Taxote
0	0	55	1	1	0	1	AC-Taxote
1	0	53	1	1	0	1	4AC-4T
0	0	55	1	1	0	3	AC
1	1	48	1	1	0	1	AC-Taxote
1	0	54	1	1	0	1	AC-Taxote
0	0	33	0	0	0	1	4AC-4T
1	1	33	0	0	0	1	4AC-4T
1	1	38	0	0	0	1	AC-Taxote
1	1	52	1	1	1	3	TCH
1	0	43	1	0	0	1	AC+monot
0	0	51	1	0	0	1	AC+monot
1	0	45	1	0	1	1	AC+monot
1	0	40	0	0	0	1	AC-Taxote
1	0	49	1	1	0	1	AC+monoT
1	0	47	1	1	0	1	AC-Taxote
0	0	46	1	0	0	1	AC-Taxote
0	0	41	1	0	1	1	AC-Taxote
1	1	57	1	1	0	1	AC+monot
0	0	64	1	1	0	1	AC-Taxote
1	0	68	1	1	0	1	AC+monot
1	1	64	1	1	1	1	AC+Taxote
1	1	43	1	0	0	1	4AC-4T
1	1	55	1	1	0	1	AC+Taxote
0	0	48	1	0	0	1	AC+monot
0	0	44	1	0	1	1	AC+monot
1	1	42	1	0	0	1	AC-Monot
1	1	51	1	0	0	1	AC-Monot

ini_cT3grou	ini_cLN2gr	HG12_Grou	Mol_Subty	ER_1percen	PR_1percen	final_HER2	PreKi_15gr
1	0	1	3	0	0	0	1
2	1	1	3	0	0	0	1
1	1	1	0	0	1	0	1
2	1	1	3	0	0	0	1
1	1	0	0	1	1	0	0
1	1	0	0	1	0	0	0
2	1	0	0	1	1	0	9
2	1	1	0	1	1	0	0
2	1	0	0	1	0	0	1
1	1	0	0	1	1	0	9
1	1	1	3	0	0	0	1
1	1	1	0	1	1	0	9
3	0	1	3	0	0	0	9
2	1	0	3	0	0	0	1
1	1	0	1	1	0	1	0
2	1	1	2	0	0	1	0
1	1	0	2	0	0	1	0
2	1	1	0	1	0	0	1
2	1	1	1	1	1	1	1
3	1	1	0	1	1	0	0
1	0	1	3	0	0	0	1
2	1	0	0	1	0	0	0
2	1	0	0	1	1	0	9
2	1	0	1	1	1	1	9
2	1	0	2	0	0	1	0
2	1	1	3	0	0	0	1
2	1	0	0	1	0	0	0
2	1	1	3	0	0	0	1
1	1	1	1	1	0	1	0
2	1	0	1	1	0	1	0
2	1	0	3	0	0	0	1
1	1	1	3	0	0	0	1
3	1	0	0	1	1	0	1
2	1	1	2	0	0	1	1
1	1	0	3	0	0	0	1
3	0	1	3	0	0	0	1
1	1	1	3	0	0	0	1
2	1	1	0	1	0	0	1
2	0	0	3	0	0	0	1
1	1	0	0	1	1	0	1
2	1	1	3	0	0	0	1
2	1	0	2	0	0	1	0

1	1	1	0	1	0	0	1
1	1	1	3	0	0	0	1
3	1	0	0	1	1	0	0
3	0	0	0	1	0	0	0
2	1	1	2	0	0	1	1
2	1	0	1	1	1	1	0
2	1	0	2	0	0	1	1
1	1	1	2	0	0	1	9
2	1	1	1	1	0	1	1
2	1	1	3	0	0	0	1
2	1	0	0	1	1	0	0
2	1	0	3	0	0	0	1
3	1	0	1	1	1	1	1
1	1	0	0	1	1	0	1
1	1	1	2	0	0	1	0
1	1	0	0	1	1	0	0
1	1	1	3	0	0	0	1
2	1	0	0	1	1	0	0
2	1	1	1	1	0	1	9
1	1	1	2	0	0	1	9
1	1	0	0	1	1	0	0
2	1	1	2	0	0	1	0
1	1	1	3	0	0	0	1
2	1	0	2	0	0	1	1
2	1	1	3	0	0	0	1
1	1	0	3	0	0	0	9
3	1	1	3	0	0	0	0
2	1	0	3	0	0	0	9
2	0	0	3	0	0	0	0
2	1	0	1	1	0	1	1
2	1	1	0	1	0	0	1
2	1	0	3	0	0	0	1
1	1	1	2	0	0	1	0
2	1	1	3	0	0	0	1
1	0	0	3	0	0	0	1
1	1	0	2	0	0	1	0
2	1	0	2	0	0	1	1
1	1	0	1	1	1	1	0
1	1	0	2	0	0	1	1
2	1	0	3	0	0	0	1
3	1	0	3	0	0	0	1
1	1	0	1	1	1	1	1
2	1	0	1	1	0	1	1

3	1	1	2	0	0	1	1
2	0	0	2	0	0	1	1
1	1	0	0	1	1	0	1
2	1	0	2	0	0	1	1
2	1	0	3	0	0	0	1
1	1	1	3	0	0	0	1
2	0	0	3	0	0	0	0
2	1	1	2	0	0	1	1
2	1	0	1	1	0	1	0
2	1	1	0	1	0	0	0
2	0	0	1	1	0	1	1
2	0	1	3	0	0	0	1
3	1	1	1	1	0	1	0
3	1	1	2	0	0	1	9
2	1	0	0	1	1	0	1
1	0	0	0	1	0	0	1
1	1	0	1	1	0	1	1
2	1	0	3	0	0	0	1
2	1	0	0	1	0	0	0
1	1	1	3	0	0	0	1
2	1	0	0	1	1	0	0
2	1	0	0	1	1	0	0
2	1	0	1	1	0	1	1
1	1	1	3	0	0	0	1
2	1	1	2	0	0	1	1
3	1	1	2	0	0	1	1
3	0	1	2	0	0	1	1
2	1	0	3	0	0	0	0
1	1	0	0	1	1	0	0
2	1	0	3	0	0	0	1
2	1	1	1	1	0	1	0
2	1	1	2	0	0	1	1

OpName_(	PRE_1_MM	PRE_1_US	PRE_1_MRI
0	0.1875	0.25	0.253968
0	0.517647	0.25	0.62963
0	0.517647	0.506173	0.62963
1	0.517647	0.506173	0.62963
0	0.1875	0.506173	0.253968
1	0.517647	0.506173	0.62963
0	0.517647	0.506173	0.62963
1	0.517647	0.506173	0.62963
0	0.517647	0.506173	0.62963
0	0.1875	0.25	0.253968
0	0.1875	0.25	0.62963
1	0.517647	0.25	0.62963
0	0.517647	0.506173	0.253968
0	0.517647	0.506173	0.253968
0	0.517647	0.506173	0.253968
0	0.517647	0.25	0.253968
1	0.517647	0.506173	0.62963
1	0.517647	0.506173	0.62963
0	0.517647	0.506173	0.62963
1	0.517647	0.506173	0.62963
0	0.517647	0.506173	0.62963
0	0.517647	0.506173	0.253968
0	0.517647	0.506173	0.62963
1	0.517647	0.506173	0.62963
1	0.517647	0.506173	0.253968
0	0.517647	0.506173	0.62963
0	0.517647	0.25	0.253968
1	0.517647	0.506173	0.62963
0	0.1875	0.506173	0.253968
1	0.517647	0.506173	0.62963
1	0.517647	0.506173	0.62963
1	0.517647	0.25	0.62963
1	0.517647	0.25	0.62963
1	0.517647	0.506173	0.62963
0	0.1875	0.25	0.253968
0	0.517647	0.506173	0.62963
1	0.517647	0.506173	0.62963
1	0.517647	0.506173	0.62963
0	0.517647	0.506173	0.62963
0	0.1875	0.506173	0.253968
0	0.1875	0.25	0.253968
0	0.517647	0.506173	0.62963

0	0.517647	0.506173	0.62963
0	0.1875	0.25	0.253968
1	0.1875	0.25	0.253968
1	0.517647	0.25	0.62963
1	0.517647	0.506173	0.253968
0	0.517647	0.506173	0.62963
1	0.1875	0.506173	0.253968
1	0.517647	0.506173	0.253968
0	0.517647	0.25	0.253968
1	0.1875	0.506173	0.253968
1	0.1875	0.25	0.253968
0	0.517647	0.506173	0.62963
1	0.517647	0.25	0.62963
1	0.517647	0.25	0.253968
0	0.517647	0.506173	0.253968
0	0.517647	0.506173	0.253968
0	0.1875	0.506173	0.253968
1	0.517647	0.506173	0.253968
0	0.517647	0.506173	0.62963
1	0.1875	0.506173	0.253968
1	0.517647	0.506173	0.62963
1	0.517647	0.506173	0.62963
0	0.1875	0.506173	0.253968
1	0.517647	0.25	0.62963
0	0.517647	0.506173	0.253968
0	0.1875	0.25	0.253968
1	0.517647	0.506173	0.62963
0	0.517647	0.506173	0.253968
1	0.517647	0.506173	0.62963
1	0.517647	0.506173	0.253968
1	0.1875	0.25	0.253968
0	0.1875	0.25	0.253968
0	0.517647	0.506173	0.62963
0	0.1875	0.25	0.253968
0	0.517647	0.25	0.253968
0	0.1875	0.506173	0.253968
0	0.517647	0.506173	0.62963
1	0.517647	0.506173	0.253968
0	0.517647	0.506173	0.253968
0	0.517647	0.506173	0.253968
1	0.517647	0.506173	0.62963
0	0.517647	0.25	0.253968
0	0.1875	0.506173	0.253968

1	0.517647	0.25	0.253968
0	0.517647	0.506173	0.62963
0	0.517647	0.25	0.253968
0	0.517647	0.506173	0.253968
0	0.517647	0.506173	0.62963
0	0.1875	0.25	0.253968
0	0.517647	0.506173	0.253968
0	0.1875	0.25	0.253968
1	0.517647	0.506173	0.62963
0	0.517647	0.506173	0.253968
0	0.1875	0.25	0.253968
0	0.517647	0.506173	0.62963
1	0.517647	0.506173	0.62963
1	0.517647	0.506173	0.62963
0	0.517647	0.506173	0.253968
1	0.1875	0.25	0.253968
0	0.1875	0.506173	0.253968
1	0.517647	0.506173	0.253968
0	0.517647	0.506173	0.253968
0	0.1875	0.506173	0.253968
0	0.1875	0.25	0.253968
1	0.517647	0.25	0.253968
1	0.517647	0.506173	0.62963
0	0.1875	0.25	0.253968
0	0.1875	0.506173	0.253968
1	0.517647	0.506173	0.62963
1	0.517647	0.506173	0.62963
0	0.517647	0.506173	0.62963
1	0.517647	0.25	0.253968
0	0.517647	0.25	0.253968
1	0.517647	0.506173	0.62963
1	0.1875	0.506173	0.62963
